# Bivariate tensor product $(p, q)$-analogue of Kantorovich-type Bernstein-Stancu-Schurer operators

**DOI:** 10.1186/s13660-017-1559-9

**Published:** 2017-11-14

**Authors:** Qing-Bo Cai, Xiao-Wei Xu, Guorong Zhou

**Affiliations:** 1 School of Mathematics and Computer Science, Quanzhou Normal University, Quanzhou, 362000 China; 2 School of Mathematical Sciences, Xiamen University, Xiamen, 361005 China; 3 Computer Sciences, Rice University, Houston, USA; 4 School of Applied Mathematics, Xiamen University of Technology, Xiamen, 361024 China

**Keywords:** 41A10, 41A25, 41A36, $(p, q)$-integers, Bernstein-Stancu-Schurer operators, modulus of continuity, Lipschitz continuous functions, bivariate tensor product

## Abstract

In this paper, we construct a bivariate tensor product generalization of Kantorovich-type Bernstein-Stancu-Schurer operators based on the concept of $(p, q)$-integers. We obtain moments and central moments of these operators, give the rate of convergence by using the complete modulus of continuity for the bivariate case and estimate a convergence theorem for the Lipschitz continuous functions. We also give some graphs and numerical examples to illustrate the convergence properties of these operators to certain functions.

## Introduction

In recent years, $(p, q)$-integers have been introduced to linear positive operators to construct new approximation processes. A sequence of $(p, q)$-analogue of Bernstein operators was first introduced by Mursaleen [1, 2]. Besides, $(p, q)$-analogues of Szász-Mirakyan operators [3], Baskakov-Kantorovich operators [4], Bleimann-Butzer-Hahn operators [5] and Kantorovich-type Bernstein-Stancu-Schurer operators [6] were also considered. For further developments, one can also refer to [7–12]. These operators are double parameters corresponding to *p* and *q* versus single parameter *q*-type operators [13–16]. The aim of these generalizations is to provide appropriate and powerful tools to application areas such as numerical analysis, CAGD and solutions of differential equations (see, e.g., [17]).

Motivated by all the above results, in 2016, Cai et al. [6] introduced a new kind of Kantorovich-type Bernstein-Stancu-Schurer operators based on $(p, q)$-integers as follows:

1 $$ K_{n,p,q}^{\alpha,\beta,l}(f;x)=\bigl([n+1]_{p,q}+\beta\bigr)\sum _{k=0}^{n+l}\frac {b_{n+l,k}(p,q;x)}{[k+1]_{p,q}-[k]_{p,q}} \int_{\frac{[k]_{p,q}+\alpha }{[n+1]_{p,q}+\beta}}^{\frac{[k+1]_{p,q}+\alpha}{[n+1]_{p,q}+\beta }}f(t)\, d_{p,q}t, $$

where $b_{n+l,k}(p,q;x)=\bigl [ {\scriptsize\begin{matrix}{} n+l\cr k \end{matrix}} \bigr ]_{p,q}x^{k}(1-x)_{p,q}^{n+l-k} $ for $f\in C(I)$, $I=[0,1+l]$, $l\in\mathbb{N}$, $0\leq\alpha\leq\beta$, $0< q< p\leq1$ and $n\in\mathbb{N}$. They got some approximation properties, since convergence properties of bivariate operators are important in approximation theory, and it seems there has been no papers mentioning the bivariate forms of above operators (1). Hence, we will propose the bivariate case in the following. Before doing this, in [6] (Lemma 2.1), they got $K_{n,p,q}^{\alpha,\beta,l}(1;x)=1$, that is, the operators reproduce constant functions. However, this conclusion is incorrect. In fact, $\sum_{k=0}^{n+l}b_{n+l,k}(p,q;x)\neq1$. Hence, we re-introduce the revised operators as

2 $$ K_{n,p,q}^{\alpha,\beta,l}(f;x)=\bigl([n+1]_{p,q}+\beta\bigr)\sum _{k=0}^{n+l}\frac {\widetilde{b_{n+l,k}}(p,q;x)}{[k+1]_{p,q}-[k]_{p,q}} \int_{\frac {[k]_{p,q}+\alpha}{[n+1]_{p,q}+\beta}}^{\frac{[k+1]_{p,q}+\alpha }{[n+1]_{p,q}+\beta}}f(t)\, d_{p,q}t, $$

where

3 $$ \widetilde{b_{n+l,k}}(p,q;x)=\frac{1}{p^{\frac{(n+l)(n+l-1)}{2}}}\left [ \textstyle\begin{array}{@{}c@{}} n+l\\ k \end{array}\displaystyle \right ]_{p,q}p^{\frac{k(k-1)}{2}}x^{k}(1-x)_{p,q}^{n+l-k}. $$

From [2], we know $\sum_{k=0}^{n+l}\widetilde {b_{n+l,k}}(p,q;x)=1$, and this ensures the operators reproduce constant functions.

On this basis, let $C(I^{2})$ denote the space of all real-valued continuous functions on $I^{2}$ endowed with the norm $\|f\| _{I^{2}}=\sup_{(x,y)\in I^{2}}|f(x,y)|$. For $f\in C(I^{2})$, $I^{2}=I\times I=[0,1+l]\times[0,1+l]$, $l\in\mathbb{N}$, $0\leq\alpha\leq\beta$, $0< q_{n_{1}}, q_{n_{2}}< p_{n_{1}}, p_{n_{2}}\leq1$ and $n_{1}, n_{2}\in\mathbb {N}$. We propose the bivariate tensor product $(p, q)$-analogue of Kantorovich-type Bernstein-Stancu-Schurer operators as follows:

4 $$\begin{aligned}& {K_{p_{n_{1}},q_{n_{1}},p_{n_{2}},q_{n_{2}}}^{n_{1},n_{2},\alpha,\beta ,l}}(f;x,y) \\& \quad = \bigl([n_{1}+1]_{p_{n_{1}},q_{n_{1}}}+\beta \bigr) \bigl([n_{2}+1]_{p_{n_{2}},q_{n_{2}}}+\beta \bigr) \\& \qquad {}\times\sum_{k_{1}=0}^{n_{1}+l}\sum _{k_{2}=0}^{n_{2}+l}\frac {b_{n_{1}+l,n_{2}+l,k_{1},k_{2}}^{p_{n_{1}},q_{n_{1}},p_{n_{2}},q_{n_{2}}}(x,y)}{ ([k_{1}+1]_{p_{n_{1}},q_{n_{1}}}-[k_{1}]_{p_{n_{1}},q_{n_{1}}} ) ([k_{2}+1]_{p_{n_{2}},q_{n_{2}}}-[k_{2}]_{p_{n_{2}},q_{n_{2}}} )} \\& \qquad {}\times \int_{\frac{[k_{1}]_{p_{n_{1}},q_{n_{1}}}+\alpha }{[n_{1}+1]_{p_{n_{1}},q_{n_{1}}}+\beta}}^{\frac {[k_{1}+1]_{p_{n_{1}},q_{n_{1}}}+\alpha}{[n_{1}+1]_{p_{n_{1}},q_{n_{1}}}+\beta}} \int _{\frac{[k_{2}]_{p_{n_{2}},q_{n_{2}}}+\alpha}{[n_{2}+1]_{p_{n_{2}},q_{n_{2}}}+\beta }}^{\frac{[k_{2}+1]_{p_{n_{2}},q_{n_{2}}}+\alpha }{[n_{2}+1]_{p_{n_{2}},q_{n_{2}}}+\beta }}f(t,s)\, d_{p_{n_{1}},q_{n_{1}}}t\, d_{p_{n_{2}},q_{n_{2}}}s, \end{aligned}$$

where

5 $$\begin{aligned}& b_{n_{1}+l,n_{2}+l,k_{1},k_{2}}^{p_{n_{1}},q_{n_{1}},p_{n_{2}},q_{n_{2}}}(x,y) \\& \quad =\frac{1}{p_{n_{1}}^{\frac{(n_{1}+l)(n_{1}+l-1)}{2}}p_{n_{2}}^{\frac {(n_{2}+l)(n_{2}+l-1)}{2}}}\left [ \textstyle\begin{array}{@{}c@{}} n_{1}+l\\ k_{1} \end{array}\displaystyle \right ]_{p_{n_{1}},q_{n_{1}}}\left [ \textstyle\begin{array}{@{}c@{}} n_{2}+l\\ k_{2} \end{array}\displaystyle \right ]_{p_{n_{2}},q_{n_{2}}} \\& \qquad {}\times p_{n_{1}}^{\frac{k_{1}(k_{1}-1)}{2}}p_{n_{2}}^{\frac {k_{2}(k_{2}-1)}{2}}x^{k_{1}}y^{k_{2}}(1-x)_{p_{n_{1}},q_{n_{1}}}^{n_{1}+l-k_{1}}(1-y)_{p_{n_{2}},q_{n_{2}}}^{n_{2}+l-k_{2}} \end{aligned}$$

for $x, y\in[0,1]$.

The paper is organized as follows. The following section contains some basic definitions regarding $(p, q)$-integers and $(p, q)$-calculus. In Section 3, we estimate the moments and central moments of the revised operators (2) and then deduce the corresponding results of a bivariate case. In Section 4, we give the rate of convergence by using the modulus of continuity and estimate a convergent theorem for the Lipschitz continuous functions. In Section 5, we give some graphs and numerical examples to illustrate the convergence properties of operators (4) to certain functions.

## Some notations

We mention some definitions based on $(p, q)$-integers, details can be found in [18–22]. For any fixed real number $0< q< p\leq 1$ and each nonnegative integer *k*, we denote $(p, q)$-integers by $[k]_{p,q}$, where

$$ [k]_{p,q}=\frac{p^{k}-q^{k}}{p-q}. $$

Also $(p, q)$-factorial and $(p, q)$-binomial coefficients are defined as follows:

$$\begin{aligned}& [k]_{p,q}!= \textstyle\begin{cases} [k]_{p,q}[k-1]_{p,q}\cdots[1]_{p,q}, &k=1,2,\ldots, \\ 1, &k=0, \end{cases}\displaystyle \\& \left [ \textstyle\begin{array}{@{}c@{}} n\\ k \end{array}\displaystyle \right ]_{p,q}= \frac{[n]_{p,q}!}{[k]_{p,q}![n-k]_{p,q}!}\quad (n\geq k\geq0). \end{aligned}$$

The $(p, q)$-Binomial expansion is defined by

$$ (x+y)_{p,q}^{n}= \textstyle\begin{cases} 1, &n=0, \\ (x+y)(px+qy)\cdots (p^{n-1}x+q^{n-1}y ), &n=1,2,\ldots. \end{cases} $$

The definite $(p, q)$-integrals are defined by

$$\begin{aligned}& \int_{0}^{a}f(x)\, d_{p,q}x=(p-q)a\sum _{k=0}^{\infty}\frac {q^{k}}{p^{k+1}}f \biggl( \frac{q^{k}}{p^{k+1}}a \biggr)\quad \mbox{and} \\& \int_{0}^{a_{1}} \int _{0}^{a_{2}}f(x,y)\, d_{p_{n_{1}},q_{n_{1}}}x\, d_{p_{n_{2}},q_{n_{2}}}y \\& \quad = (p_{n_{1}}-q_{n_{1}} ) (p_{n_{2}}-q_{n_{2}} )a_{1}a_{2} \sum_{k_{1}=0}^{\infty} \sum_{k_{2}=0}^{\infty}\frac {q_{n_{1}}^{k_{1}}}{p_{n_{1}}^{k_{1}+1}} \frac {q_{n_{2}}^{k_{2}}}{p_{n_{2}}^{k_{2}+1}}f \biggl(\frac {q_{n_{1}}^{k_{1}}}{p_{n_{1}}^{k_{1}+1}}a_{1}, \frac {q_{n_{2}}^{k_{2}}}{p_{n_{2}}^{k_{2}+1}}a_{2} \biggr). \end{aligned}$$

When $p=1$, all the definitions of $(p, q)$-calculus above are reduced to *q*-calculus.

## Auxiliary results

In order to obtain the convergence properties, we need the following lemmas.

### Lemma 3.1

*For the* $(p, q)$-*analogue of Kantorovich*-*type Bernstein*-*Stancu*-*Schurer operators* (2), *we have*

6 $$\begin{aligned}& {K_{n,p,q}^{\alpha,\beta,l}}(1;x)=1, \end{aligned}$$

7 $$\begin{aligned}& {K_{n,p,q}^{\alpha,\beta,l}}(t;x)=\frac {(1+q)[n+l]_{p,q}(px+1-x)_{p,q}^{n+l-1}}{[2]_{p,q} ([n+1]_{p,q}+\beta )p^{n+l-1}}x +\frac{(px+1-x)_{p,q}^{n+l}+2\alpha}{[2]_{p,q} ([n+1]_{p,q}+\beta )} , \end{aligned}$$

8 $$\begin{aligned}& {K_{n,p,q}^{\alpha,\beta,l}} \bigl(t^{2};x \bigr) \\& \quad = \frac{ (q+q^{2}+q^{3} )[n+l]_{p,q}[n+l-1]_{p,q} (p^{2}x+1-x )_{p,q}^{n+l-2}}{[3]_{p,q} ([n+1]_{p,q}+\beta )^{2}p^{2n+2l-4}}x^{2} \\& \qquad{} +\frac{ (1+q+q^{2}+p+2pq )[n+l]_{p,q} (p^{2}x+1-x )_{p,q}^{n+l-1}x}{[3]_{p,q} ([n+1]_{p,q}+\beta )^{2}p^{n+l-2}} \\& \qquad {}+\frac{3\alpha(1+q)[n+l]_{p,q}(px+1-x)_{p,q}^{n+l-1}x}{[3]_{p,q} ([n+1]_{p,q}+\beta )^{2}p^{n+l-1}}+\frac{ (p^{2}x+1-x )_{p,q}^{n+l}}{[3]_{p,q} ([n+1]_{p,q}+\beta )^{2}} \\& \qquad {}+\frac{3\alpha (px+1-x )_{p,q}^{n+l}+3{\alpha }^{2}}{[3]_{p,q} ([n+1]_{p,q}+\beta )^{2}}. \end{aligned}$$

### Proof

Since $\sum_{k=0}^{n+l}{b_{n+l,k}}(p,q;x)=1$, (6) is easily obtained. Using (2) and $[k+1]_{p,q}=p^{k}+q[k]_{p,q}$, we have

$$\begin{aligned}& {K_{n,p,q}^{\alpha,\beta,l}}(t;x) \\& \quad = \bigl([n+1]_{p,q}+\beta\bigr)\sum_{k=0}^{n+l} \frac {b_{n+l,k}(p,q;x)}{[k+1]_{p,q}-[k]_{p,q}} \int_{\frac{[k]_{p,q}+\alpha }{[n+1]_{p,q}+\beta}}^{\frac{[k+1]_{p,q}+\alpha}{[n+1]_{p,q}+\beta }}t\, d_{p,q}t \\& \quad = \bigl([n+1]_{p,q}+\beta\bigr)\sum_{k=0}^{n+l} \frac {b_{n+l,k}(p,q;x)}{[k+1]_{p,q}-[k]_{p,q}}\frac{([k+1]_{p,q}+\alpha )^{2}-([k]_{p,q}+\alpha)^{2}}{[2]_{p,q}([n+1]_{p,q}+\beta)^{2}} \\& \quad = \frac{1}{[2]_{p,q}([n+1]_{p,q}+\beta)}\sum_{k=0}^{n+l}b_{n+l,k}(p,q;x) \bigl([k+1]_{p,q}+[k]_{p,q}+2\alpha\bigr) \\& \quad = \frac{(1+q)[n+l]_{p,q}x}{[2]_{p,q} ([n+1]_{p,q}+\beta )p^{n+l-1}p^{\frac{(n+l-1)(n+l-2)}{2}}}\sum_{k=0}^{n+l-1} \left [ \textstyle\begin{array}{@{}c@{}} n+l-1 \\ k \end{array}\displaystyle \right ]_{p,q}p^{\frac{k(k-1)}{2}} \\& \qquad {}\times(px)^{k}(1-x)_{p,q}^{n+l-k-1}+ \frac{(px+1-x)_{p,q}^{n+l}+2\alpha }{[2]_{p,q} ([n+1]_{p,q}+\beta )} \\& \quad = \frac{(1+q)[n+l]_{p,q}(px+1-x)_{p,q}^{n+l-1}}{[2]_{p,q} ([n+1]_{p,q}+\beta )p^{n+l-1}}x+\frac{(px+1-x)_{p,q}^{n+l}+2\alpha }{[2]_{p,q} ([n+1]_{p,q}+\beta )}. \end{aligned}$$

Thus, (7) is proved. Finally, from (2), we get

$$\begin{aligned}& {K_{n,p,q}^{\alpha,\beta}} \bigl(t^{2};x \bigr) \\& \quad = \bigl([n+1]_{p,q}+\beta\bigr)\sum_{k=0}^{n+l} \frac {b_{n+l,k}(p,q;x)}{[k+1]_{p,q}-[k]_{p,q}} \int_{\frac{[k]_{p,q}+\alpha }{[n+1]_{p,q}+\beta}}^{\frac{[k+1]_{p,q}+\alpha}{[n+1]_{p,q}+\beta }}t^{2}\, d_{p,q}t \\& \quad = \frac{ ([n+1]_{p,q}+\beta )}{[3]_{p,q}}\sum_{k=0}^{n+l} \frac {b_{n+l,k}(p,q;x)}{[k+1]_{p,q}-[k]_{p,q}}\frac{([k+1]_{p,q}+\alpha )^{3}-([k]_{p,q}+\alpha)^{3}}{([n+1]_{p,q}+\beta)^{3}} \\& \quad = \frac{1}{[3]_{p,q}([n+1]_{p,q}+\beta)^{2}}\sum_{k=0}^{n+l} \bigl([k+1]_{p,q}^{2}+[k]_{p,q}^{2}+[k+1]_{p,q}[k]_{p,q} \\& \qquad {} +3\alpha[k+1]_{p,q}+3\alpha[k]_{p,q}+3{ \alpha}^{2}\bigr)b_{n+l,k}(p,q;x). \end{aligned}$$

Since $[k+1]_{p,q}=p^{k}+q[k]_{p,q}$, by some computations, we get

$$\begin{aligned} \begin{aligned} &[k+1]_{p,q}^{2}+[k]_{p,q}^{2}+[k+1]_{p,q}[k]_{p,q}+3 \alpha [k+1]_{p,q}+3\alpha[k]_{p,q}+3{\alpha}^{2} \\ &\quad = \bigl(q+q^{2}+q^{3} \bigr)[k]_{p,q}[k-1]_{p,q}+ \biggl(1+2q+\frac {1+q+q^{2}}{p} \biggr)p^{k}[k]_{p,q} \\ &\qquad {}+3\alpha (1+q)[k]_{p,q}+p^{2k}+3\alpha p^{k}+3{\alpha}^{2}. \end{aligned} \end{aligned}$$

So, we can obtain

$$\begin{aligned}& {K_{n,p,q}^{\alpha,\beta,l}} \bigl(t^{2};x \bigr) \\& \quad = \frac{ (q+q^{2}+q^{3} )[n+l]_{p,q}[n+l-1]_{p,q} (p^{2}x+1-x )_{p,q}^{n+l-2}}{[3]_{p,q} ([n+1]_{p,q}+\beta )^{2}p^{2n+2l-4}}x^{2} \\& \qquad {} +\frac{ (1+q+q^{2}+p+2pq )[n+l]_{p,q} (p^{2}x+1-x )_{p,q}^{n+l-1}x}{[3]_{p,q} ([n+1]_{p,q}+\beta )^{2}p^{n+l-2}} \\& \qquad {} +\frac{3\alpha(1+q)[n+l]_{p,q}(px+1-x)_{p,q}^{n+l-1}x}{[3]_{p,q} ([n+1]_{p,q}+\beta )^{2}p^{n+l-1}}+\frac{ (p^{2}x+1-x )_{p,q}^{n+l}}{[3]_{p,q} ([n+1]_{p,q}+\beta )^{2}} \\& \qquad {} +\frac{3\alpha (px+1-x )_{p,q}^{n+l}+3{\alpha }^{2}}{[3]_{p,q} ([n+1]_{p,q}+\beta )^{2}}. \end{aligned}$$

Thus, (8) is proved. □

### Lemma 3.2

*Using Lemma* 3.1 *and easy computations*, *we have*

9 $$\begin{aligned}& K_{n,p,q}^{\alpha,\beta,l}(t-x;x) \\& \quad = \biggl(\frac{(1+q)[n+l]_{p,q}(px+1-x)_{p,q}^{n+l-1}}{[2]_{p,q} ([n+1]_{p,q}+\beta )p^{n+l-1}}-1 \biggr)x+\frac {(px+1-x)_{p,q}^{n+l}+2\alpha}{[2]_{p,q} ([n+1]_{p,q}+\beta )}, \end{aligned}$$

10 $$\begin{aligned}& K_{n,p,q}^{\alpha,\beta,l} \bigl((t-x)^{2};x \bigr) \\& \quad = \biggl(\frac{ (q+q^{2}+q^{3} )[n+l]_{p,q}[n+l-1]_{p,q} (p^{2}x+1-x )_{p,q}^{n+l-2}}{[3]_{p,q} ([n+1]_{p,q}+\beta )^{2}p^{2n+2l-4}}+1 \\& \qquad {} -\frac{2(1+q)[n+l]_{p,q} (px+1-x )_{p,q}^{n+l-1}}{[2]_{p,q} ([n+l]_{p,q}+\beta )p^{n+l-1}} \biggr)x^{2}+\frac{ (p^{2}x+1-x )_{p,q}^{n+l}}{[3]_{p,q} ([n+1]_{p,q}+\beta )^{2}} \\& \qquad {}+\frac{ (1+q+q^{2}+p+2pq )[n+l]_{p,q} (p^{2}x+1-x )_{p,q}^{n+l-1}x}{[3]_{p,q} ([n+1]_{p,q}+\beta )^{2}p^{n+l-2}} \\& \qquad {}+\frac{3\alpha(1+q)[n+l]_{p,q}(px+1-x)_{p,q}^{n+l-1}x}{[3]_{p,q} ([n+1]_{p,q}+\beta )^{2}p^{n+l-1}}+\frac{3\alpha (px+1-x)_{p,q}^{n+l}+3{\alpha}^{2}}{[3]_{p,q} ([n+1]_{p,q}+\beta )^{2}} \\& \qquad {}-\frac{2(px+1-x)_{p,q}^{n+l}x+4\alpha x}{[2]_{p,q} ([n+1]_{p,q}+\beta )}. \end{aligned}$$

### Lemma 3.3

*Let* $e_{i,j}(x,y)=x^{i}y^{j}$, $i,j\in\mathbb{N}$, $i+j\leq2$, $(x,y)\in I^{2}$ *be the two*-*dimensional test functions*. *Using Lemma* 3.1, *the bivariate* $(p, q)$-*analogue of Kantorovich*-*type Bernstein*-*Stancu*-*Schurer operators defined in* (4) *satisfies the following equalities*:

11 $$\begin{aligned}& K_{p_{n_{1}},q_{n_{1}},p_{n_{2}},q_{n_{2}}}^{n_{1},n_{2},\alpha,\beta ,l}(e_{0,0};x,y)=1, \end{aligned}$$

12 $$\begin{aligned}& K_{p_{n_{1}},q_{n_{1}},p_{n_{2}},q_{n_{2}}}^{n_{1},n_{2},\alpha,\beta ,l}(e_{1,0};x,y) \\ & \quad =\frac{(p_{n_{1}}x+1-x)_{p_{n_{1}},q_{n_{1}}}^{n_{1}+l}+2\alpha }{[2]_{p_{n_{1}},q_{n_{1}}} ([n_{1}+1]_{p_{n_{1}},q_{n_{1}}}+\beta )}+\frac {(1+q_{n_{1}})[n_{1}+l]_{p_{n_{1}},q_{n_{1}}}(p_{n_{1}}x+1-x)_{p_{n_{1}},q_{n_{1}}}^{n_{1}+l-1}}{[2]_{p_{n_{1}},q_{n_{1}}} ([n_{1}+1]_{p_{n_{1}},q_{n_{1}}}+\beta )p_{n_{1}}^{n_{1}+l-1}}x, \end{aligned}$$

13 $$\begin{aligned}& K_{p_{n_{1}},q_{n_{1}},p_{n_{2}},q_{n_{2}}}^{n_{1},n_{2},\alpha,\beta ,l}(e_{0,1};x,y) \\ & \quad =\frac{(p_{n_{2}}y+1-y)_{p_{n_{2}},q_{n_{2}}}^{n_{2}+l}+2\alpha }{[2]_{p_{n_{2}},q_{n_{2}}} ([n_{2}+1]_{p_{n_{2}},q_{n_{2}}}+\beta )}+\frac {(1+q_{n_{2}})[n_{2}+l]_{p_{n_{2}},q_{n_{2}}}(p_{n_{2}}y+1-y)_{p_{n_{2}},q_{n_{2}}}^{n_{2}+l-1}}{[2]_{p_{n_{2}},q_{n_{2}}} ([n_{2}+1]_{p_{n_{2}},q_{n_{2}}}+\beta )p_{n_{2}}^{n_{2}+l-1}}y, \end{aligned}$$

14 $$\begin{aligned}& K_{p_{n_{1}},q_{n_{1}},p_{n_{2}},q_{n_{2}}}^{n_{1},n_{2},\alpha,\beta ,l}(e_{1,1};x,y) \\ & \quad =\biggl(\frac {(p_{n_{1}}x+1-x)_{p_{n_{1}},q_{n_{1}}}^{n_{1}+l}+2\alpha }{[2]_{p_{n_{1}},q_{n_{1}}} ([n_{1}+1]_{p_{n_{1}},q_{n_{1}}}+\beta )}+\frac {(1+q_{n_{1}})[n_{1}+l]_{p_{n_{1}},q_{n_{1}}}(p_{n_{1}}x+1-x)_{p_{n_{1}},q_{n_{1}}}^{n_{1}+l-1}}{[2]_{p_{n_{1}},q_{n_{1}}} ([n_{1}+1]_{p_{n_{1}},q_{n_{1}}}+\beta )p_{n_{1}}^{n_{1}+l-1}}x\biggr) \\ & \qquad {}\times\biggl(\frac {(1+q_{n_{2}})[n_{2}+l]_{p_{n_{2}},q_{n_{2}}}(p_{n_{2}}y+1-y)_{p_{n_{2}},q_{n_{2}}}^{n_{2}+l-1}}{[2]_{p_{n_{2}},q_{n_{2}}} ([n_{2}+1]_{p_{n_{2}},q_{n_{2}}}+\beta )p_{n_{2}}^{n_{2}+l-1}}y \\ & \qquad {}+\frac{(p_{n_{2}}y+1-y)_{p_{n_{2}},q_{n_{2}}}^{n_{2}+l}+2\alpha }{[2]_{p_{n_{2}},q_{n_{2}}} ([n_{2}+1]_{p_{n_{2}},q_{n_{2}}}+\beta )}\biggr), \end{aligned}$$

15 $$\begin{aligned}& K_{p_{n_{1}},q_{n_{1}},p_{n_{2}},q_{n_{2}}}^{n_{1},n_{2},\alpha,\beta ,l}(e_{2,0};x,y) \\ & \quad = \frac{ (q_{n_{1}}+q_{n_{1}}^{2}+q_{n_{1}}^{3} )[n_{1}+l]_{p_{n_{1}},q_{n_{1}}}[n_{1}+l-1]_{p_{n_{1}},q_{n_{1}}} (p_{n_{1}}^{2}x+1-x )_{p_{n_{1}},q_{n_{1}}}^{n_{1}+l-2}}{[3]_{p_{n_{1}},q_{n_{1}}} ([n_{1}+1]_{p_{n_{1}},q_{n_{1}}}+\beta )^{2}p_{n_{1}}^{2n_{1}+2l-4}}x^{2} \\ & \qquad {}+\frac{ (1+q_{n_{1}}+q_{n_{1}}^{2}+p_{n_{1}}+2p_{n_{1}}q_{n_{1}} )[n_{1}+l]_{p_{n_{1}},q_{n_{1}}} (p_{n_{1}}^{2}x+1-x )_{p_{n_{1}},q_{n_{1}}}^{n_{1}+l-1}x}{[3]_{p_{n_{1}},q_{n_{1}}} ([n_{1}+1]_{p_{n_{1}},q_{n_{1}}}+\beta )^{2}p_{n_{1}}^{n_{1}+l-2}} \\ & \qquad {}+\frac{3\alpha (1+q_{n_{1}})[n_{1}+l]_{p_{n_{1}},q_{n_{1}}}(p_{n_{1}}x+1-x)_{p_{n_{1}},q_{n_{1}}}^{n_{1}+l-1}x}{[3]_{p_{n_{1}},q_{n_{1}}} ([n_{1}+1]_{p_{n_{1}},q_{n_{1}}}+\beta )^{2}p_{n_{1}}^{n_{1}+l-1}} \\ & \qquad {}+\frac{ (p_{n_{1}}^{2}x+1-x )_{p_{n_{1}},q_{n_{1}}}^{n_{1}+l}}{[3]_{p_{n_{1}},q_{n_{1}}} ([n_{1}+1]_{p_{n_{1}},q_{n_{1}}}+\beta )^{2}}+\frac{3\alpha (p_{n_{1}}x+1-x )_{p_{n_{1}},q_{n_{1}}}^{n_{1}+l}+3{\alpha }^{2}}{[3]_{p_{n_{1}},q_{n_{1}}} ([n_{1}+1]_{p_{n_{1}},q_{n_{1}}}+\beta )^{2}}, \end{aligned}$$

16 $$\begin{aligned}& K_{p_{n_{1}},q_{n_{1}},p_{n_{2}},q_{n_{2}}}^{n_{1},n_{2},\alpha,\beta ,l}(e_{0,2};x,y) \\ & \quad = \frac{ (q_{n_{2}}+q_{n_{2}}^{2}+q_{n_{2}}^{3} )[n_{2}+l]_{p_{n_{2}},q_{n_{2}}}[n_{2}+l-1]_{p_{n_{2}},q_{n_{2}}} (p_{n_{2}}^{2}y+1-y )_{p_{n_{2}},q_{n_{2}}}^{n_{2}+l-2}}{[3]_{p_{n_{2}},q_{n_{2}}} ([n_{2}+1]_{p_{n_{2}},q_{n_{2}}}+\beta )^{2}p_{n_{2}}^{2n_{2}+2l-4}}y^{2} \\ & \qquad {}+\frac{ (1+q_{n_{2}}+q_{n_{2}}^{2}+p_{n_{2}}+2p_{n_{2}}q_{n_{2}} )[n_{2}+l]_{p_{n_{2}},q_{n_{2}}} (p_{n_{2}}^{2}y+1-y )_{p_{n_{2}},q_{n_{2}}}^{n_{2}+l-1}y}{[3]_{p_{n_{2}},q_{n_{2}}} ([n_{2}+1]_{p_{n_{2}},q_{n_{2}}}+\beta )^{2}p_{n_{2}}^{n_{2}+l-2}} \\ & \qquad {}+\frac{3\alpha (1+q_{n_{2}})[n_{2}+l]_{p_{n_{2}},q_{n_{2}}}(p_{n_{2}}y+1-y)_{p_{n_{2}},q_{n_{2}}}^{n_{2}+l-1}y}{[3]_{p_{n_{2}},q_{n_{2}}} ([n_{2}+1]_{p_{n_{2}},q_{n_{2}}}+\beta )^{2}p_{n_{2}}^{n_{2}+l-1}} \\ & \qquad {}+\frac{ (p_{n_{2}}^{2}y+1-y )_{p_{n_{2}},q_{n_{2}}}^{n_{2}+l}}{[3]_{p_{n_{2}},q_{n_{2}}} ([n_{2}+1]_{p_{n_{2}},q_{n_{2}}}+\beta )^{2}}+\frac{3\alpha (p_{n_{2}}y+1-y )_{p_{n_{2}},q_{n_{2}}}^{n_{2}+l}+3{\alpha }^{2}}{[3]_{p_{n_{2}},q_{n_{2}}} ([n_{2}+1]_{p_{n_{2}},q_{n_{2}}}+\beta )^{2}}. \end{aligned}$$

### Lemma 3.4

*Using Lemmas* 3.2 *and* 3.3, *the following equalities hold*:

17 $$\begin{aligned}& K_{p_{n_{1}},q_{n_{1}},p_{n_{2}},q_{n_{2}}}^{n_{1},n_{2},\alpha,\beta ,l}(t-x;x,y) \\& \quad = \biggl(\frac {(1+q_{n_{1}})[n_{1}+l]_{p_{n_{1}},q_{n_{1}}}(p_{n_{1}}x+1-x)_{p_{n_{1}},q_{n_{1}}}^{n_{1}+l-1}}{[2]_{p_{n_{1}},q_{n_{1}}} ([n_{1}+1]_{p_{n_{1}},q_{n_{1}}}+\beta )p_{n_{1}}^{n_{1}+l-1}}-1 \biggr)x \\& \qquad {}+\frac{(p_{n_{1}}x+1-x)_{p_{n_{1}},q_{n_{1}}}^{n_{1}+l}+2\alpha }{[2]_{p_{n_{1}},q_{n_{1}}} ([n_{1}+1]_{p_{n_{1}},q_{n_{1}}}+\beta )}:=A_{n_{1},p_{n_{1}},q_{n_{1}}}^{\alpha,\beta,l}(x), \end{aligned}$$

18 $$\begin{aligned}& K_{p_{n_{1}},q_{n_{1}},p_{n_{2}},q_{n_{2}}}^{n_{1},n_{2},\alpha,\beta ,l}(s-y;x,y)=A_{n_{2},p_{n_{2}},q_{n_{2}}}^{\alpha,\beta,l}(y), \end{aligned}$$

19 $$\begin{aligned}& K_{p_{n_{1}},q_{n_{1}},p_{n_{2}},q_{n_{2}}}^{n_{1},n_{2},\alpha,\beta,l} \bigl((t-x)^{2};x,y \bigr) \\& \quad = \biggl(\frac{ (q_{n_{1}}+q_{n_{1}}^{2}+q_{n_{1}}^{3} )[n_{1}+l]_{p_{n_{1}},q_{n_{1}}}[n_{1}+l-1]_{p_{n_{1}},q_{n_{1}}} (p_{n_{1}}^{2}x+1-x )_{p_{n_{1}},q_{n_{1}}}^{n_{1}+l-2}}{[3]_{p_{n_{1}},q_{n_{1}}} ([n_{1}+1]_{p_{n_{1}},q_{n_{1}}}+\beta )^{2}p_{n_{1}}^{2n_{1}+2l-4}} \\& \qquad {}+1-\frac{2(1+q_{n_{1}})[n_{1}+l]_{p_{n_{1}},q_{n_{1}}} (p_{n_{1}}x+1-x )_{p_{n_{1}},q_{n_{1}}}^{n_{1}+l-1}}{[2]_{p_{n_{1}},q_{n_{1}}} ([n_{1}+l]_{p_{n_{1}},q_{n_{1}}}+\beta )p_{n_{1}}^{n_{1}+l-1}} \biggr)x^{2} \\& \qquad {} +\frac{ (1+q_{n_{1}}+q_{n_{1}}^{2}+p_{n_{1}}+2p_{n_{1}}q_{n_{1}} )[n_{1}+l]_{p_{n_{1}},q_{n_{1}}} (p_{n_{1}}^{2}x+1-x )_{p_{n_{1}},q_{n_{1}}}^{n_{1}+l-1}x}{[3]_{p_{n_{1}},q_{n_{1}}} ([n_{1}+1]_{p_{n_{1}},q_{n_{1}}}+\beta )^{2}p_{n_{1}}^{n_{1}+l-2}} \\& \qquad {}+\frac{3\alpha (1+q_{n_{1}})[n_{1}+l]_{p_{n_{1}},q_{n_{1}}}(p_{n_{1}}x+1-x)_{p_{n_{1}},q_{n_{1}}}^{n_{1}+l-1}x}{[3]_{p_{n_{1}},q_{n_{1}}} ([n_{1}+1]_{p_{n_{1}},q_{n_{1}}}+\beta )^{2}p_{n_{1}}^{n_{1}+l-1}} \\& \qquad {}+\frac{ (p_{n_{1}}^{2}x+1-x )_{p_{n_{1}},q_{n_{1}}}^{n_{1}+l}}{[3]_{p_{n_{1}},q_{n_{1}}} ([n_{1}+1]_{p_{n_{1}},q_{n_{1}}}+\beta )^{2}}+\frac{3\alpha (p_{n_{1}}x+1-x)_{p_{n_{1}},q_{n_{1}}}^{n_{1}+l}+3{\alpha }^{2}}{[3]_{p_{n_{1}},q_{n_{1}}} ([n_{1}+1]_{p_{n_{1}},q_{n_{1}}}+\beta )^{2}} \\& \qquad {}-\frac{2(p_{n_{1}}x+1-x)_{p_{n_{1}},q_{n_{1}}}^{n_{1}+l}x+4\alpha x}{[2]_{p_{n_{1}},q_{n_{1}}} ([n_{1}+1]_{p_{n_{1}},q_{n_{1}}}+\beta )}:=B_{n_{1},p_{n_{1}},q_{n_{1}}}^{\alpha,\beta,l}(x), \end{aligned}$$

20 $$\begin{aligned}& K_{p_{n_{1}},q_{n_{1}},p_{n_{2}},q_{n_{2}}}^{n_{1},n_{2},\alpha,\beta,l} \bigl((s-y)^{2};x,y \bigr)=B_{n_{2},p_{n_{2}},q_{n_{2}}}^{\alpha,\beta,l}(y). \end{aligned}$$

### Lemma 3.5

(see Theorem 2.1 of [23])

*For* $0< q_{n}< p_{n}\leq1$, *set* $q_{n}:=1-\alpha_{n}$, $p_{n}:=1-\beta _{n}$ *such that* $0\leq\beta_{n}<\alpha_{n}<1$, $\alpha_{n}\rightarrow0$, $\beta _{n}\rightarrow0$ *as* $n\rightarrow\infty$. *The following statements are true*:

(A) *If* $\lim_{n\rightarrow\infty}e^{n(\beta_{n}-\alpha_{n})}=1$ *and* $e^{n\beta_{n}}/n\rightarrow0$, *then* $[n]_{p_{n},q_{n}}\rightarrow\infty$.
(B) *If* $\varlimsup_{n\rightarrow\infty}e^{n(\beta_{n}-\alpha _{n})}<1$ *and* $e^{n\beta_{n}}(\alpha_{n}-\beta_{n})\rightarrow0$, *then* $[n]_{p_{n},q_{n}}\rightarrow\infty$.
(C) *If* $\varliminf_{n\rightarrow\infty}e^{n(\beta_{n}-\alpha _{n})}<1$, $\varlimsup_{n\rightarrow\infty}e^{n(\beta_{n}-\alpha_{n})}=1$ *and* $\max \{e^{n\beta_{n}}/n,e^{n\beta_{n}}(\alpha_{n}-\beta_{n}) \} \rightarrow0$, *then* $[n]_{p_{n},q_{n}}\rightarrow\infty$.

### Remark 3.6

Let sequences $\{p_{n_{1}}\}$, $\{q_{n_{1}}\}$, $\{p_{n_{2}}\}$, $\{ q_{n_{2}}\}$ ($0< q_{n_{1}}, q_{n_{2}}< p_{n_{1}}, p_{n_{2}}\leq1$) satisfy the conditions of Lemma 3.5(A), (B) or (C). We have $[n_{1}]_{p_{n_{1}},q_{n_{1}}}\rightarrow\infty$, $[n_{2}]_{p_{n_{2}},q_{n_{2}}}\rightarrow\infty$. From Lemmas 3.3 and 3.4, the following statements are true.

$$\begin{aligned}& \lim_{n_{1},n_{2}\rightarrow\infty }{K_{p_{n_{1}},q_{n_{1}},p_{n_{2}},q_{n_{2}}}^{n_{1},n_{2},\alpha,\beta,l} (e_{1,0};x,y )}=x, \\& \lim_{n_{1},n_{2}\rightarrow\infty }{K_{p_{n_{1}},q_{n_{1}},p_{n_{2}},q_{n_{2}}}^{n_{1},n_{2},\alpha,\beta,l} (e_{0,1};x,y )}=y, \\& \lim_{n_{1},n_{2}\rightarrow\infty }{K_{p_{n_{1}},q_{n_{1}},p_{n_{2}},q_{n_{2}}}^{n_{1},n_{2},\alpha,\beta,l} (e_{2,0}+e_{0,2};x,y )}=x^{2}+y^{2}, \\& \lim_{n_{1},n_{2}\rightarrow\infty }{K_{p_{n_{1}},q_{n_{1}},p_{n_{2}},q_{n_{2}}}^{n_{1},n_{2},\alpha,\beta,l} \bigl((t-x)^{2};x,y \bigr)}=\lim_{n_{1}\rightarrow\infty }B_{n_{1},p_{n_{1}},q_{n_{1}}}^{\alpha,\beta,l}(x)=0, \\& \lim_{n_{1},n_{2}\rightarrow\infty }{K_{p_{n_{1}},q_{n_{1}},p_{n_{2}},q_{n_{2}}}^{n_{1},n_{2},\alpha,\beta,l} \bigl((s-y)^{2};x,y \bigr)}=\lim_{n_{2}\rightarrow\infty }B_{n_{2},p_{n_{2}},q_{n_{2}}}^{\alpha,\beta,l}(y)=0. \end{aligned}$$

## Convergence properties

In order to ensure the convergence of operators defined in (4), in the sequel, let $\{p_{n_{1}}\}$, $\{q_{n_{1}}\}$, $\{p_{n_{2}}\}$, $\{q_{n_{2}}\} $, $0< q_{n_{1}}, q_{n_{2}}< p_{n_{1}}, p_{n_{2}}\leq1$ be sequences satisfying Lemma 3.5(A), (B) or (C).

### Theorem 4.1

*For* $f\in C(I^{2})$, *we have*

$$ \lim_{n_{1},n_{2}\rightarrow\infty} \bigl\Vert K_{p_{n_{1}},q_{n_{1}},p_{n_{2}},q_{n_{2}}}^{n_{1},n_{2},\alpha,\beta,l}(f; \cdot ,\cdot)-f \bigr\Vert _{I^{2}}=0. $$

### Proof

Using (6), Remark 3.6 and a bivariate-type Korovkin theorem (see [24]), we obtain Theorem 4.1 easily. □

For $f\in C(I^{2})$, the complete modulus of continuity for the bivariate case is defined as

$$\begin{aligned}& \omega(f;\delta_{1},\delta_{2}) \\& \quad = \sup\bigl\{ \bigl\vert f(t,s)-f(x,y) \bigr\vert : (t,s), (x,y) \in I^{2}, |t-x|\leq\delta_{1}, |s-y|\leq\delta_{2} \bigr\} , \end{aligned}$$

where $\delta_{1}, \delta_{2}>0$. Furthermore, $\omega(f;\delta_{1},\delta _{2})$ satisfies the following properties:

$$\begin{aligned} (\mathrm{i})&\quad \omega(f;\delta_{1},\delta_{2}) \rightarrow0,\quad \mbox{if }\delta_{1}, \delta _{2} \rightarrow0; \\ (\mathrm{ii})&\quad \bigl|f(t,s)-f(x,y)\bigr|\leq\omega(f;\delta_{1},\delta) \biggl(1+\frac {|t-x|}{\delta_{1}} \biggr) \biggl(\frac{|s-y|}{\delta_{2}} \biggr). \end{aligned}$$

The partial modulus of continuity with respect to *x* and *y* is defined as

$$\begin{aligned}& \omega^{(1)}(f;\delta)=\sup\bigl\{ \bigl\vert f(x_{1},y)-f(x_{2},y) \bigr\vert : y\in I\mbox{ and }\vert x_{1}-x_{2} \vert \leq \delta\bigr\} , \\& \omega^{(2)}(f;\delta)=\sup\bigl\{ \bigl\vert f(x,y_{1})-f(x,y_{2}) \bigr\vert : x\in I\mbox{ and }\vert y_{1}-y_{2} \vert \leq \delta\bigr\} . \end{aligned}$$

Details of the modulus of continuity for the bivariate case can be found in [25]. We also use the notation

$$ C^{1}\bigl(I^{2}\bigr)=\bigl\{ f\in C\bigl(I^{2} \bigr): f_{x}', f_{y}'\in C \bigl(I^{2}\bigr)\bigr\} . $$

Now, we give the estimate of the rate of convergence of operators defined in (4).

### Theorem 4.2

*For* $f\in C(I^{2})$, *we have*

21 $$ \bigl\vert K_{p_{n_{1}},q_{n_{1}},p_{n_{2}},q_{n_{2}}}^{n_{1},n_{2},\alpha,\beta ,l}(f;x,y)-f(x,y) \bigr\vert \leq4 \omega \Bigl(f;\sqrt {B_{n_{1},p_{n_{1}},q_{n_{1}}}^{\alpha,\beta,l}(x)}, \sqrt {B_{n_{2},p_{n_{2}},q_{n_{2}}}^{\alpha,\beta,l}(y)} \Bigr), $$

*where* $B_{n_{1},p_{n_{1}},q_{n_{1}}}^{\alpha,\beta,l}(x)$ *and* $B_{n_{2},p_{n_{2}},q_{n_{2}}}^{\alpha,\beta,l}(y)$ *are defined in* (19) *and* (20).

### Proof

From Lemmas 3.3 and 3.4, using the property (ii) of the complete modulus of continuity for the bivariate case above and the Cauchy-Schwarz inequality, we get

$$\begin{aligned}& \bigl\vert K_{p_{n_{1}},q_{n_{1}},p_{n_{2}},q_{n_{2}}}^{n_{1},n_{2},\alpha,\beta ,l}(f;x,y)-f(x,y) \bigr\vert \\& \quad \leq K_{p_{n_{1}},q_{n_{1}},p_{n_{2}},q_{n_{2}}}^{n_{1},n_{2},\alpha,\beta ,l}\bigl( \bigl\vert f(t,s)-f(x,y) \bigr\vert ;x,y\bigr) \\& \quad \leq \omega \Bigl(f;\sqrt{B_{n_{1},p_{n_{1}},q_{n_{1}}}^{\alpha,\beta ,l}(x)}, \sqrt{B_{n_{2},p_{n_{2}},q_{n_{2}}}^{\alpha,\beta,l}(y)} \Bigr) \\& \qquad {} \times \biggl(1+\sqrt{\frac {K_{p_{n_{1}},q_{n_{1}},p_{n_{2}},q_{n_{2}}}^{n_{1},n_{2},\alpha,\beta,l} ((t-x)^{2};x,y )}{B_{n_{1},p_{n_{1}},q_{n_{1}}}^{\alpha,\beta,l}(x)}} \biggr) \\& \qquad {} \times \biggl(1+\sqrt{\frac {K_{p_{n_{1}},q_{n_{1}},p_{n_{2}},q_{n_{2}}}^{n_{1},n_{2},\alpha,\beta,l} ((s-y)^{2};x,y )}{B_{n_{2},p_{n_{2}},q_{n_{2}}}^{\alpha,\beta,l}(y)}} \biggr). \end{aligned}$$

Theorem 4.2 is proved. □

### Theorem 4.3

*For* $f\in C(I^{2})$, *under the conditions of Lemma* 3.4, *we have*

$$\begin{aligned}& \bigl\vert K_{p_{n_{1}},q_{n_{1}},p_{n_{2}},q_{n_{2}}}^{n_{1},n_{2},\alpha,\beta ,l}(f;x,y)-f(x,y) \bigr\vert \\& \quad \leq 2 \Bigl(f;\omega^{(1)} \Bigl(f;\sqrt{B_{n_{1},p_{n_{1}},q_{n_{1}}}^{\alpha ,\beta,l}(x)} \Bigr)+\omega^{(2)} \Bigl(f;\sqrt {B_{n_{2},p_{n_{2}},q_{n_{2}}}^{\alpha,\beta,l}(y)} \Bigr) \Bigr), \end{aligned}$$

*where* $B_{n_{1},p_{n_{1}},q_{n_{1}}}^{\alpha,\beta,l}(x)$ *and* $B_{n_{2},p_{n_{2}},q_{n_{2}}}^{\alpha,\beta,l}(y)$ *are defined in* (19) *and* (20).

### Proof

Using the definition of partial modulus of continuity above and the Cauchy-Schwarz inequality, we have

$$\begin{aligned}& \bigl\vert K_{p_{n_{1}},q_{n_{1}},p_{n_{2}},q_{n_{2}}}^{n_{1},n_{2},\alpha,\beta ,l}(f;x,y)-f(x,y) \bigr\vert \\& \quad \leq K_{p_{n_{1}},q_{n_{1}},p_{n_{2}},q_{n_{2}}}^{n_{1},n_{2},\alpha,\beta ,l}\bigl( \bigl\vert f(t,s)-f(x,y) \bigr\vert ;x,y\bigr) \\& \quad \leq K_{p_{n_{1}},q_{n_{1}},p_{n_{2}},q_{n_{2}}}^{n_{1},n_{2},\alpha,\beta ,l}\bigl( \bigl\vert f(t,s)-f(t,y) \bigr\vert ;x,y\bigr) \\& \qquad {}+K_{p_{n_{1}},q_{n_{1}},p_{n_{2}},q_{n_{2}}}^{n_{1},n_{2},\alpha,\beta ,l}\bigl( \bigl\vert f(t,y)-f(x,y) \bigr\vert ;x,y\bigr) \\& \quad \leq K_{p_{n_{1}},q_{n_{1}},p_{n_{2}},q_{n_{2}}}^{n_{1},n_{2},\alpha,\beta,l} \bigl(\omega^{(2)}\bigl(f; \vert s-y \vert \bigr);x,y \bigr) \\& \qquad {}+K_{p_{n_{1}},q_{n_{1}},p_{n_{2}},q_{n_{2}}}^{n_{1},n_{2},\alpha,\beta,l} \bigl(\omega^{(1)}\bigl(f; \vert t-x \vert \bigr);x,y \bigr) \\& \quad \leq \omega^{(2)} \Bigl(f;\sqrt{B_{n_{2},p_{n_{2}},q_{n_{2}}}^{\alpha,\beta ,l}(y)} \Bigr) \biggl(1+\sqrt{\frac {K_{p_{n_{1}},q_{n_{1}},p_{n_{2}},q_{n_{2}}}^{n_{1},n_{2},\alpha,\beta,l} ((s-y)^{2};x,y )}{B_{n_{2},p_{n_{2}},q_{n_{2}}}^{\alpha,\beta,l}(y)}} \biggr) \\& \qquad {}+\omega^{(1)} \Bigl(f;\sqrt{B_{n_{1},p_{n_{1}},q_{n_{1}}}^{\alpha,\beta ,l}(x)} \Bigr) \biggl(1+\sqrt{\frac {K_{p_{n_{1}},q_{n_{1}},p_{n_{2}},q_{n_{2}}}^{n_{1},n_{2},\alpha,\beta,l} ((t-x)^{2};x,y )}{B_{n_{1},p_{n_{1}},q_{n_{1}}}^{\alpha,\beta,l}(x)}} \biggr). \end{aligned}$$

Theorem 4.3 is proved. □

### Theorem 4.4

*For* $f\in C^{1}(I^{2})$, *using Lemma* 3.4, *we have*

$$\begin{aligned}& \bigl\vert K_{p_{n_{1}},q_{n_{1}},p_{n_{2}},q_{n_{2}}}^{n_{1},n_{2},\alpha,\beta ,l}(f;x,y)-f(x,y) \bigr\vert \\& \quad \leq \bigl\Vert f_{x}' \bigr\Vert _{I}\sqrt{B_{n_{1},p_{n_{1}},q_{n_{1}}}^{\alpha,\beta,l}(x)} + \bigl\Vert f_{y}' \bigr\Vert _{I}\sqrt {B_{n_{2},p_{n_{2}},q_{n_{2}}}^{\alpha,\beta,l}(y)}, \end{aligned}$$

*where* $B_{n_{1},p_{n_{1}},q_{n_{1}}}^{\alpha,\beta,l}(x)$ *and* $B_{n_{2},p_{n_{2}},q_{n_{2}}}^{\alpha,\beta,l}(y)$ *are defined in* (19) *and* (20).

### Proof

Since $f(t,s)-f(x,y)=\int_{x}^{t}f_{u}'(u,s)\,du+\int_{y}^{s}f_{v}'(x,v)\,dv$. Applying the operators defined in (4) on both sides above, we have

$$\begin{aligned}& \bigl\vert K_{p_{n_{1}},q_{n_{1}},p_{n_{2}},q_{n_{2}}}^{n_{1},n_{2},\alpha,\beta ,l}(f;x,y)-f(x,y) \bigr\vert \\& \quad \leq K_{p_{n_{1}},q_{n_{1}},p_{n_{2}},q_{n_{2}}}^{n_{1},n_{2},\alpha,\beta,l} \biggl( \biggl\vert \int_{x}^{t}f_{u}'(u,s) \,du \biggr\vert ;x,y \biggr) \\& \qquad {} +K_{p_{n_{1}},q_{n_{1}},p_{n_{2}},q_{n_{2}}}^{n_{1},n_{2},\alpha,\beta,l} \biggl( \biggl\vert \int_{y}^{s}f_{v}'(x,v) \,dv \biggr\vert ;x,y \biggr). \end{aligned}$$

Due to $\vert \int_{x}^{t}f_{u}'(u,s)\,du \vert \leq\|f_{x}'\|_{I}|t-x|$ and $\vert \int_{y}^{s}f_{v}'(x,v)\,dv \vert \leq\|f_{y}'\|_{I}|s-y|$, we have

$$\begin{aligned} &\bigl\vert K_{p_{n_{1}},q_{n_{1}},p_{n_{2}},q_{n_{2}}}^{n_{1},n_{2},\alpha,\beta ,l}(f;x,y)-f(x,y) \bigr\vert \\ &\quad \leq \bigl\Vert f_{x}' \bigr\Vert _{I}K_{p_{n_{1}},q_{n_{1}},p_{n_{2}},q_{n_{2}}}^{n_{1},n_{2},\alpha ,\beta,l}\bigl( \vert t-x \vert ;x,y \bigr)+ \bigl\Vert f_{y}' \bigr\Vert _{I}K_{p_{n_{1}},q_{n_{1}},p_{n_{2}},q_{n_{2}}}^{n_{1},n_{2},\alpha,\beta,l}\bigl( \vert s-y \vert ;x,y \bigr). \end{aligned}$$

Using the Cauchy-Schwarz inequality and Lemma 3.3, we obtain

$$\begin{aligned}& \bigl\vert K_{p_{n_{1}},q_{n_{1}},p_{n_{2}},q_{n_{2}}}^{n_{1},n_{2},\alpha,\beta ,l}(f;x,y)-f(x,y) \bigr\vert \\& \quad \leq \bigl\Vert f_{x}' \bigr\Vert _{I}\sqrt {K_{p_{n_{1}},q_{n_{1}},p_{n_{2}},q_{n_{2}}}^{n_{1},n_{2},\alpha,\beta,l} \bigl((t-x)^{2};x,y \bigr)}\sqrt {K_{p_{n_{1}},q_{n_{1}},p_{n_{2}},q_{n_{2}}}^{n_{1},n_{2},\alpha,\beta,l}(1;x,y)} \\& \qquad {} + \bigl\Vert f_{y}' \bigr\Vert _{I}\sqrt {K_{p_{n_{1}},q_{n_{1}},p_{n_{2}},q_{n_{2}}}^{n_{1},n_{2},\alpha,\beta,l} \bigl((s-y)^{2};x,y \bigr)}\sqrt {K_{p_{n_{1}},q_{n_{1}},p_{n_{2}},q_{n_{2}}}^{n_{1},n_{2},\alpha,\beta,l}(1;x,y)} \\& \quad \leq \bigl\Vert f_{x}' \bigr\Vert _{I}\sqrt{B_{n_{1},p_{n_{1}},q_{n_{1}}}^{\alpha,\beta ,l}(x)}+ \bigl\Vert f_{y}' \bigr\Vert _{I}\sqrt {B_{n_{2},p_{n_{2}},q_{n_{2}}}^{\alpha,\beta,l}(y)}. \end{aligned}$$

Theorem 4.4 is proved. □

Finally, we study the rate of convergence of $K_{p_{n_{1}},q_{n_{1}},p_{n_{2}},q_{n_{2}}}^{n_{1},n_{2},\alpha,\beta,l}(f;x,y)$ by means of functions of Lipschitz class $\operatorname{Lip}_{M}(\theta_{1},\theta_{2})$ if

$$ \bigl\vert f(t,s)-f(x,y) \bigr\vert \leq M|t-x|^{\theta_{1}}|s-y|^{\theta_{2}}, \quad (t,s), (x,y)\in I^{2}. $$

### Theorem 4.5

*Let* $f\in \operatorname{Lip}_{M}(\theta_{1},\theta_{2})$, *under the conditions of Lemma* 3.4, *we have*

$$ \bigl\vert K_{p_{n_{1}},q_{n_{1}},p_{n_{2}},q_{n_{2}}}^{n_{1},n_{2},\alpha,\beta ,l}(f;x,y)-f(x,y) \bigr\vert \leq M \bigl(B_{n_{1},p_{n_{1}},q_{n_{1}}}^{\alpha ,\beta,l}(x) \bigr)^{\frac{\theta_{1}}{2}} \bigl(B_{n_{2},p_{n_{2}},q_{n_{2}}}^{\alpha,\beta,l}(y) \bigr)^{\frac{\theta_{2}}{2}}, $$

*where* $B_{n_{1},p_{n_{1}},q_{n_{1}}}^{\alpha,\beta,l}(x)$ *and* $B_{n_{2},p_{n_{2}},q_{n_{2}}}^{\alpha,\beta,l}(y)$ *are defined in* (19) *and* (20).

### Proof

Since $f\in \operatorname{Lip}_{M}(\theta_{1},\theta_{2})$, we have

$$\begin{aligned}& \bigl\vert K_{p_{n_{1}},q_{n_{1}},p_{n_{2}},q_{n_{2}}}^{n_{1},n_{2},\alpha,\beta ,l}(f;x,y)-f(x,y) \bigr\vert \\& \quad \leq K_{p_{n_{1}},q_{n_{1}},p_{n_{2}},q_{n_{2}}}^{n_{1},n_{2},\alpha,\beta ,l}\bigl( \bigl\vert f(t,s)-f(x,y) \bigr\vert ;x,y\bigr) \\& \quad \leq MK_{p_{n_{1}},q_{n_{1}},p_{n_{2}},q_{n_{2}}}^{n_{1},n_{2},\alpha,\beta,l} \bigl( \vert t-x \vert ^{\theta_{1}};x,y \bigr)K_{p_{n_{1}},q_{n_{1}},p_{n_{2}},q_{n_{2}}}^{n_{1},n_{2},\alpha,\beta,l} \bigl( \vert s-y \vert ^{\theta_{2}};x,y \bigr). \end{aligned}$$

Using Hölder’s inequality for the last formula, respectively, we get

$$\begin{aligned}& \bigl\vert K_{p_{n_{1}},q_{n_{1}},p_{n_{2}},q_{n_{2}}}^{n_{1},n_{2},\alpha,\beta ,l}(f;x,y)-f(x,y) \bigr\vert \\& \quad \leq M \bigl(K_{p_{n_{1}},q_{n_{1}},p_{n_{2}},q_{n_{2}}}^{n_{1},n_{2},\alpha,\beta ,l}\bigl((t-x)^{2};x,y \bigr) \bigr)^{\frac{\theta_{1}}{2}} \bigl(K_{p_{n_{1}},q_{n_{1}},p_{n_{2}},q_{n_{2}}}^{n_{1},n_{2},\alpha,\beta ,l}(1;x,y) \bigr)^{\frac{2-\theta_{1}}{2}} \\& \qquad {} \times \bigl(K_{p_{n_{1}},q_{n_{1}},p_{n_{2}},q_{n_{2}}}^{n_{1},n_{2},\alpha,\beta ,l}\bigl((s-y)^{2};x,y \bigr) \bigr)^{\frac{\theta_{2}}{2}} \bigl(K_{p_{n_{1}},q_{n_{1}},p_{n_{2}},q_{n_{2}}}^{n_{1},n_{2},\alpha,\beta ,l}(1;x,y) \bigr)^{\frac{2-\theta_{2}}{2}} \\& \quad = M \bigl(B_{n_{1},p_{n_{1}},q_{n_{1}}}^{\alpha,\beta,l}(x) \bigr)^{\frac {\theta_{1}}{2}} \bigl(B_{n_{2},p_{n_{2}},q_{n_{2}}}^{\alpha,\beta,l}(y) \bigr)^{\frac{\theta_{2}}{2}}. \end{aligned}$$

Theorem 4.5 is proved. □

## Graphical and numerical examples analysis

In this section, we give several graphs and numerical examples to show the convergence of $K_{p_{n_{1}},q_{n_{1}},p_{n_{2}},q_{n_{2}}}^{n_{1},n_{2},\alpha,\beta,l}(f;x,y)$ to $f(x,y)$ with different values of parameters which satisfy the conclusions of Lemma 3.5.

**Figure 1:**
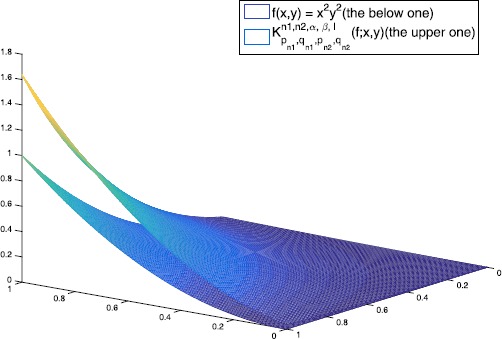
**The figures of** $\pmb{K_{p_{n_{1}},q_{n_{1}},p_{n_{2}},q_{n_{2}}}^{n_{1},n_{2},\alpha,\beta,l}(f;x,y)}$ **(the upper one) for** $\pmb{n_{1} = n_{2} = 20}$ **,** $\pmb{p_{n_{1}} = p_{n_{2}} = 1 - 1/10^{8}}$ **,** $\pmb{q_{n_{1}} = q_{n_{2}} = 0.999}$ **,** $\pmb{l=1}$ **,** $\pmb{\alpha=3}$ **,** $\pmb{\beta=2}$ **and** $\pmb{f(x,y)=x^{2}y^{2}}$ **(the below one).**

**Figure 2:**
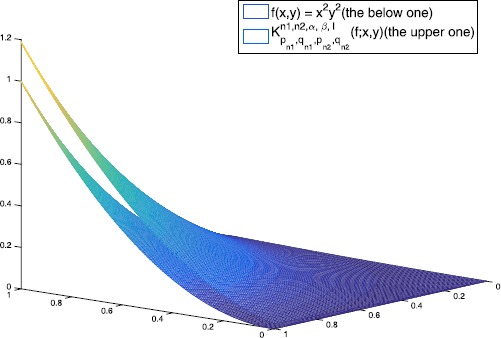
**The figures of** $\pmb{K_{p_{n_{1}},q_{n_{1}},p_{n_{2}},q_{n_{2}}}^{n_{1},n_{2},\alpha,\beta,l}(f;x,y)}$ **(the upper one) for** $\pmb{n_{1} = n_{2} = 20}$ **,** $\pmb{p_{n_{1}} = p_{n_{2}} = 1 - 1/10^{14}}$ **,** $\pmb{q_{n_{1}} = q_{n_{2}} = 0.9999}$ **,** $\pmb{l=1}$ **,** $\pmb{\alpha=3}$ **,** $\pmb{\beta=2}$ **and** $\pmb{f(x,y)=x^{2}y^{2}}$ **(the below one).**

**Figure 3:**
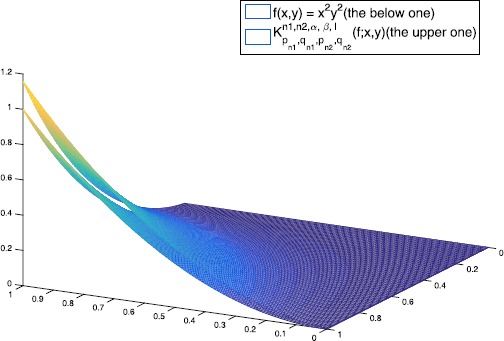
**The figures of** $\pmb{K_{p_{n_{1}},q_{n_{1}},p_{n_{2}},q_{n_{2}}}^{n_{1},n_{2},\alpha,\beta,l}(f;x,y)}$ **(the upper one) for** $\pmb{n_{1} = n_{2} = 50}$ **,** $\pmb{p_{n_{1}} = p_{n_{2}} = 1 - 1/10^{14}}$ **,** $\pmb{q_{n_{1}} = q_{n_{2}} = 0.9999}$ **,** $\pmb{l=1}$ **,** $\pmb{\alpha=3}$ **,** $\pmb{\beta=2}$ **and** $\pmb{f(x,y)=x^{2}y^{2}}$ **(the below one).**

Let $f(x,y) = x^{2}y^{2}$, the graphs of $K_{p_{n_{1}},q_{n_{1}},p_{n_{2}},q_{n_{2}}}^{n_{1},n_{2},\alpha,\beta,l}(f;x,y)$ with different values of $q_{n_{1}}$, $q_{n_{2}}$, $p_{n_{1}}$, $p_{n_{2}}$ and $n_{1}$, $n_{2}$ are shown in Figures 1, 2 and 3. In Tables 1, 2 and 3, we give the errors of the approximation of $K_{p_{n_{1}},q_{n_{1}},p_{n_{2}},q_{n_{2}}}^{n_{1},n_{2},\alpha,\beta,l}(f;x,y)$ to $f(x,y)$ with different parameters.

**Table 1:** **The errors of the approximation of** $\pmb{K_{p_{n_{1}},q_{n_{1}},p_{n_{2}},q_{n_{2}}}^{n_{1},n_{2},\alpha,\beta,l}(f;x,y)}$ **for** $\pmb{p_{n_{1}} = p_{n_{2}} = 1{-}1/10^{15}}$ **,** $\pmb{q_{n_{1}} = q_{n_{2}} = 0.9999}$ **,** $\pmb{l=1}$ **,** $\pmb{\alpha=3}$ **,** $\pmb{\beta=2}$ **and different values of** $\pmb{n_{1}}$ **,** $\pmb{n_{2}}$

$\boldsymbol{n_{1} = n_{2}}$	$\boldsymbol{\|f(x,y) - K_{p_{n_{1}},q_{n_{1}},p_{n_{2}},q_{n_{2}}}^{n_{1},n_{2},\alpha,\beta,l}(f;x,y)\| _{\infty}}$
5	0.801911
10	0.406691
15	0.259663
20	0.188202
30	0.150588
35	0.131835

**Table 2:** **The errors of the approximation of** $\pmb{K_{p_{n_{1}},q_{n_{1}},p_{n_{2}},q_{n_{2}}}^{n_{1},n_{2},\alpha,\beta,l}(f;x,y)}$ **for** $\pmb{n_{1} = n_{2} = 10}$ **,** $\pmb{p_{n_{1}} = p_{n_{2}} = 1{-}1/10^{15}}$ **,** $\pmb{l=1}$ **,** $\pmb{\alpha=3}$ **,** $\pmb{\beta=2}$ **and different values of** $\pmb{q_{n_{1}}}$ **,** $\pmb{q_{n_{2}}}$

$\boldsymbol{q_{n_{1}} = q_{n_{2}}}$	$\boldsymbol{\|f(x,y) - K_{p_{n_{1}},q_{n_{1}},p_{n_{2}},q_{n_{2}}}^{n_{1},n_{2},\alpha,\beta,l}(f;x,y)\| _{\infty}}$
0.99	2.923910
0.995	1.194710
0.999	0.643543
0.9995	0.594722
0.9999	0.406691
0.99995	0.130489

**Table 3:** **The errors of the approximation of** $\pmb{K_{p_{n_{1}},q_{n_{1}},p_{n_{2}},q_{n_{2}}}^{n_{1},n_{2},\alpha,\beta,l}(f;x,y)}$ **for** $\pmb{q_{n_{1}} = q_{n_{2}} = 0.9999}$ **,** $\pmb{l=1}$ **,** $\pmb{\alpha=3}$ **,** $\pmb{\beta=2}$ **and different values of** $\pmb{p_{n_{1}}}$ **,** $\pmb{p_{n_{2}}}$ **and** $\pmb{n_{1}}$ **,** $\pmb{n_{2}}$

$\boldsymbol{n_{1} =n_{2}}$	$\boldsymbol{p_{n_{1}}=p_{n_{2}}}$	$\boldsymbol{\|f(x,y) - K_{p_{n_{1}},q_{n_{1}},p_{n_{2}},q_{n_{2}}}^{n_{1},n_{2},\alpha,\beta ,l}(f;x,y)\|_{\infty}}$
10	1 − 1/10^10^	0.406691
15	1 − 1/10^11^	0.259663
20	1 − 1/10^12^	0.188202
25	1 − 1/10^13^	0.150589
30	1 − 1/10^14^	0.131836
35	1 − 1/10^15^	0.125673
